# Horizontal ecological compensation and urban inclusive green growth: evidence from China

**DOI:** 10.3389/fpubh.2024.1415309

**Published:** 2024-09-24

**Authors:** Hengli Wang, Weiyi Li, Hongce Xiao, Daoli Wang

**Affiliations:** ^1^Institute of Big Data, Zhongnan University of Economics and Law, Wuhan, China; ^2^School of Mathematics and Statistics, Zhongnan University of Economics and Law, Wuhan, China

**Keywords:** horizontal ecological compensation, ecological compensation, inclusive green growth, inequitable environment, green development

## Abstract

**Introduction:**

Horizontal ecological compensation (HEC) has the potential to incentivize inclusive green growth in cities.

**Methods:**

Using the multi-stage difference-in-differences (DID) method, this study examines the impact of HEC policies as a quasi-natural experiment. Panel data are analyzed; the data pertain to 87 cities in the Yangtze River Basin, from 2007 to 2020.

**Results:**

The findings indicate that HEC policies significantly contribute to inclusive green growth, with consistent effects across different estimators. The moderating effect test reveals that urban industrial pollution levels and green innovation are key pathways through which HEC policies influence inclusive green growth. Further analysis shows that the positive impact of HEC is more pronounced in watersheds with high marketization and in downstream regions, suggesting that HEC may exacerbate regional disparities in inclusive green growth.

**Discussion:**

This study offers insights for China and also for other developing countries seeking to promote urban inclusive green growth and achieve sustainable development goals.

## 1 Introduction

The impact of ecological compensation policies on environmental governance is currently a critical topic in the field of environmental health and for those responsible for forming public policy. To effectively measure the sustainable development of the economy, society, and ecology within environmental governance, inclusive green growth (IGG) serves as a key indicator. The horizontal ecological compensation (HEC) policy remains under-researched, despite that policy’s significance as an institutional innovation aimed at fostering ecological civilization. This raises an essential question: Can China’s HEC policy promote IGG?

Since the country’s reform and opening up, China has grappled with significant issues, such as income inequality, uneven development, and environmental degradation (1–3). These challenges stem from a lack of inclusivity and environmental focus in traditional governance (4). As a critical tool in environmental governance, ecological compensation plays a crucial role in government efforts to promote environmental protection and sustainable development (5–7). Prioritizing ecological compensation policies is an essential means of guiding cities toward greener, more inclusive, and sustainable development (8).

In 2012, the academic community introduced the concept of IGG at the Rio +20 Summit (9). The IGG approach advocates for economic development that provides equal opportunities for all (10, 11) and ensures that the benefits of growth are shared by everyone in society (12). Also, IGG emphasizes social equity, environmental sustainability, and the well-being of both present and future generations (13).

Unlike inclusive growth in general or just green growth, IGG seeks a balance between the economy, society, and the environment. This growth model aims to conserve natural resources, protect the environment, and promote inclusive social development (14). By integrating economic, social, and ecological considerations, IGG fosters sustainable development across all three systems (15, 16). The horizontal ecological compensation (HEC) policy is a significant institutional innovation designed to promote an ecological civilization. To enhance the protection and restoration of ecosystems, this policy aims to establish a cooperative transfer system between local governments with close ecological ties. Unlike vertical transfer systems, the HEC is more effective in addressing fiscal equalization and externalities.

However, further study is required to ensure that HEC policies genuinely foster IGG, where environmental benefits are equitably distributed among all members of society (17). A crucial need exists to examine how the preferences and interests of government policymakers impact equity and inclusiveness when those policymakers are designing and implementing HEC policies.

To address these issues, the relationship between ecological compensation and IGG needs to be explored, with full consideration given to the actual needs of ecological protection and economic and social development. Investigating the impact mechanism of ecological compensation on IGG is essential. Research on the use of HEC to promote IGG is limited. Most existing studies focus either on the benefits of ecological compensation or on the externalities associated with IGG. Therefore, this area warrants a more comprehensive examination.

Existing literature on IGG primarily focuses on two areas. Firstly, there is the measurement of IGG. This has been explored using various methods, including the social opportunity function (18), economic complexity method (19), and the construction of indicator and evaluation systems (20, 21). Secondly, studies have investigated the impact of different factors on IGG. Recent research highlights how all of the following influence IGG: distortions in the labor market (22), the misallocation of land and finance (23, 24)), foreign direct investment (25), the digital economy (26, 27), economic development (28), green finance (29), fiscal decentralization (30), financial system reform (31), economic policy uncertainty (32), knowledge production capacity (33), and technological change and progress (34, 35).

Regarding the environmental benefits of HEC policies, some studies indicate that HEC plays a critical role in transboundary water pollution control. However, these studies also hold that HEC exacerbates conflicts between environmental protection and economic development in compensated areas. On a positive note, HEC has been found to promote labor division and collaboration, coordinate socioeconomic development with ecological protection in cities (Yi et al., 2022), improve green ecological development (36), enhance green low-carbon development (22), and adjust industrial structures (37). Conversely, Wang et al. (38) discovered that HEC indirectly inhibits technological progress in upstream companies. The study holds that HEC affects profitability, scale, human capital, foreign direct investment, and management efficiency. Therefore, there is a crucial need to examine whether HEC policies can achieve a balance between environmental and economic benefits and ultimately promote IGG.

This paper introduces an index system for inclusive green growth in 87 cities in the Yangtze River Economic Belt, covering the years from 2007 to 2020, utilizing the entropy-weighted technique for order of preference by similarity to ideal solution (TOPSIS) method. A multi-period DID model is employed to evaluate the impact of policy implementation on inclusive green growth. The study examines the effect of the HEC policy on inclusive green growth and explores the moderating roles of green innovation and urban industrial pollution levels. The findings support a thorough investigation of the relationship between IGG and HEC. The study provides guidance and recommendations for designing and implementing policies to promote both IGG and HEC in the Yangtze River Basin.

The innovations of this article are as follows:

1. Existing literature primarily focuses on the environmental improvement effects of ecological compensation. These studies neglect ecological compensation’s relationship with IGG, and thus, research on moderating mechanisms from different dimensions is lacking.
2. Evaluations of ecological compensation policies in the study area are limited to specific water areas, necessitating further research on continuous areas.
3. Most existing literature measures IGG at the provincial level, whereas this paper provides detailed data for 87 cities in the Yangtze River Economic Belt, thereby offering a research basis for studying IGG.

The remainder of this paper is structured as follows: Section 2 outlines the theoretical background and develops hypotheses on China’s HEC policy and IGG, based on existing literature. Section 3 details the empirical methodology and describes the data used in the study. Section 4 presents the empirical results, including analyses of potential pathways and robustness tests, as well as an examination of heterogeneous effects. Finally, Section 5 provides the study’s conclusions.

## 2 Theoretical analysis and hypothesis development

### 2.1 IGG and HEC policies

Inclusive green growth is essential for the coordinated development of the economy, society, and ecology (39, 40). HEC policy, a significant institutional innovation, plays a crucial role in promoting this growth. While many studies have examined the factors and effects of IGG, conclusive results have yet to be reached. To further explore the mechanisms influencing IGG, scholars have analyzed various aspects such as energy efficiency, sectoral development, and environmental regulation (41–44). Research on the impact of environmental policies on IGG has primarily focused on direct and indirect effects. HEC, an institutional arrangement that promotes ecological protection (45, 46) and social equity through economic means (47, 48), can significantly contribute to IGG by reducing pollution (49) and emissions while also alleviating individual poverty (50–52).

Dörffel and Schuhmann (53) developed an economic growth model that integrates environmental factors and human capital, highlighting the internal relationship between HEC and economic growth. Building on this, Zhen et al. (54) created a quantitative model of ecological compensation based on environmental and economic cost–benefit analyses. Their model demonstrated how ecological compensation can promote the development of ecologically intensive agriculture by calculating the internal economic costs and benefits.

Hegde and Bull (55) conducted an empirical assessment of a carbon sequestration project in Mozambique at the household level. Their findings indicated that small-scale ecological offset projects had more significant benefits for local male-headed and high-income households. However, it is important to note that these results are specific to the household level and may not be applicable to other contexts.

Several scholars have shown that HEC can aid in poverty reduction and decrease social development gaps. For instance, Zhang and Pagiola (56) found that such compensation can reduce poverty in the upper reaches of watersheds. Based on these findings, this study proposes the following hypothesis:

*H1:* HEC policies can generally increase the level of urban IGG.

### 2.2 Paths mechanisms of HEC policies on IGG

In regions implementing HEC mechanisms, the central government distributes rewards or imposes penalties based on environmental assessment outcomes. To meet these objectives, local authorities often enforce strict environmental laws. These stringent regulations pressure polluting industries to make strategic changes, such as relocating to areas with more lenient environmental regulations, reducing production, improving pollution control methods, or adopting new technologies to enhance eco-efficiency and avoid penalties (47, 48, 57).

The implementation of HEC mechanisms can effectively promote IGG. By incentivizing the conservation and restoration of ecosystems, these mechanisms support sustainable economic development and integrated ecosystem management (43). Literature indicates that ecological compensation effectively mobilizes participation in ecological protection, improves ecological environment quality, and lays a solid foundation for green development (58, 59). HEC has the potential to promote the marketization of ecological products and services, stimulate innovation in ecological protection areas, cultivate new growth points, and achieve economic transformation and upgrading (60). Furthermore, HEC enhances positive interactions between eco-protectors and beneficiaries, promotes coordinated regional development, and fosters the concept of ecological civilization through building and sharing, thus achieving IGG (61). Plenty scholars examined the environmental and economic impacts of these policies in various river basins using rigorous methods such as the double-difference method, synthetic control method, costing method, and mechanism analysis. This study provides valuable insights into the benefits and drawbacks of HEC policies (59, 62–64). The evidence from numerous studies supports the claim that ecological compensation significantly improves watershed ecological quality and promotes sustainable economic growth (48, 57, 65).

Thus, this study confidently proposes Hypothesis 2:

*H2:* The level of urban industrial pollution enhances the positive incentive effect of HEC policies on the level of IGG.

The implementation of HEC policies has encouraged enterprises to invest in environmentally friendly technologies, enhancing both green production efficiency and technological innovation (14). These policies drive industrial technological advancement by improving enterprise productivity (54). There is a mutually influential relationship between HEC policies and technological innovation. The implementation of these policies motivates enterprises and researchers to increase their investment in scientific research, thereby boosting innovation in green development and eco-governance (66), which fosters IGG propelled by technological innovation.

Recent empirical analysis by Yang (67) indicates that HEC policies can enhance scientific and technological innovation, promote industrial structure upgrades, and encourage pollution reduction and green development. This results in improved green total factor productivity (67). Additionally, Liu et al.’s (66) study on the Yangtze River Ecological Economic Zone highlights that a high-quality ecological environment, which prioritizes eco-technological innovation and eco-value realization, benefits the compensated region’s economy by achieving high-quality development.

Overall, HEC policies significantly impact green technological innovation by providing financial resources, increasing enterprise productivity, and influencing industrial structure. These effects collectively promote IGG.

Therefore, this study proposes Hypothesis 3:

*H3:* The level of green innovation enhances the positive incentive effect of HEC policies on the level of IGG.

### 2.3 Diverse regional impacts: HEC’s influence on IGG

Ecological compensation is a strategy designed to protect and conserve the environment through mutual responsibilities and benefits. The compensated party agrees to adopt strict environmental protection measures, increase the supply of ecological products, and implement higher environmental standards to prevent new pollution. In return, the compensating party requires the compensated party to forego certain economic development opportunities to provide better environmental outcomes. To offset these sacrifices, the compensating party provides financial support. This system increases the costs of ecological protection and environmental management for the compensated party and also represents a loss of potential economic growth.

**Figure 1:**
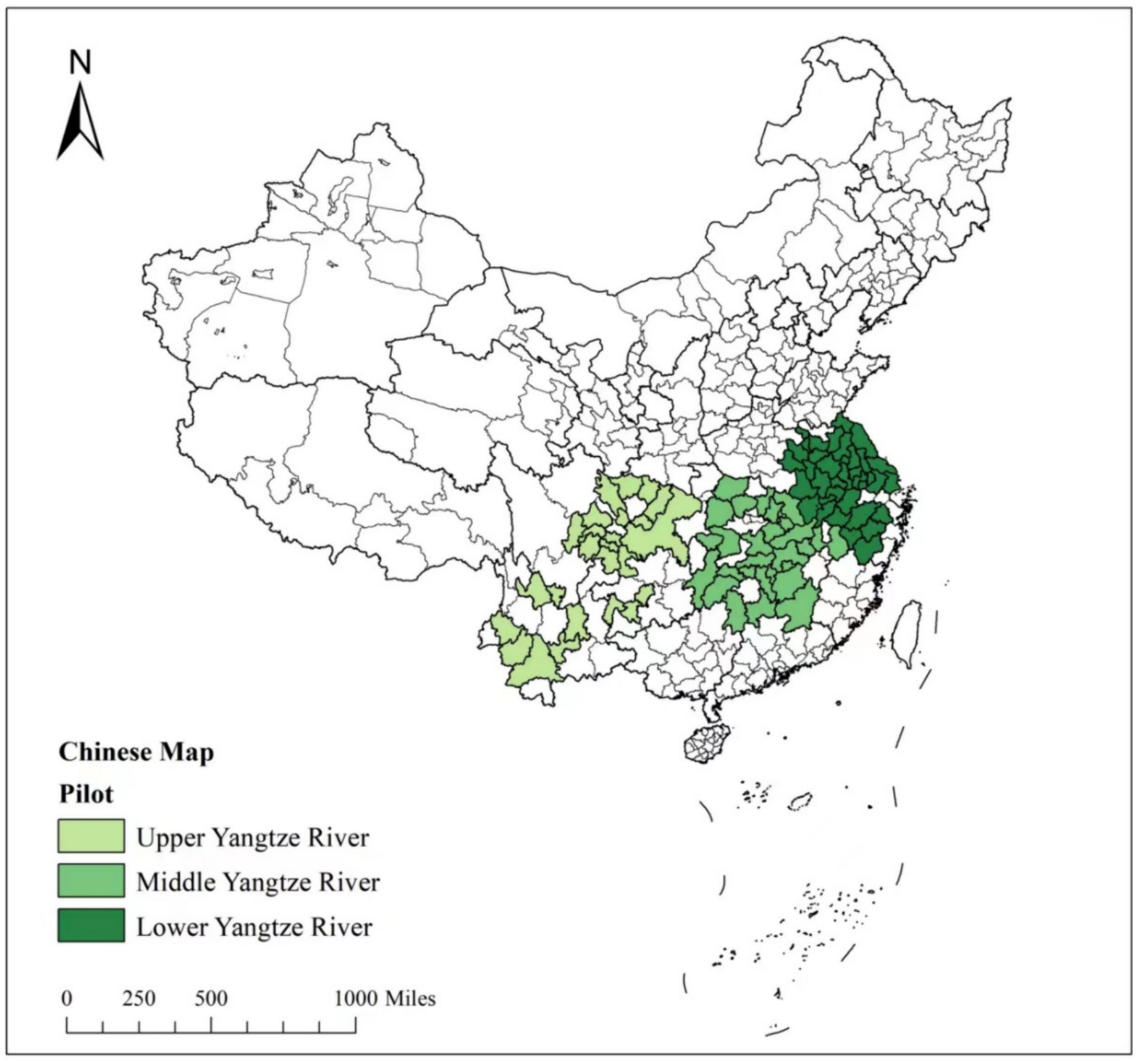
Study area.

Horizontal watershed ecological compensation specifically addresses ecological damage in the upper reaches of watersheds. These areas often have low market activity and weak economic foundations, leading to a strong demand for economic growth. This creates a significant conflict between economic development and ecological protection. Balancing these interests is crucial for sustainable development. While may raise the costs of environmental protection and limit economic opportunities in these regions, its impact on inclusive growth is limited in less marketized areas (Figure 1).

In contrast, regions with higher levels of marketization and stronger economic bases can benefit more from compensation funds. These regions can use the additional resources to foster better development opportunities and promote inclusive economic growth.

Therefore, this study proposes Hypothesis 4:

*H4:* In regions with a high degree of marketization, the effect of HEC on inclusive economic growth is more significant.

**Figure 2:**
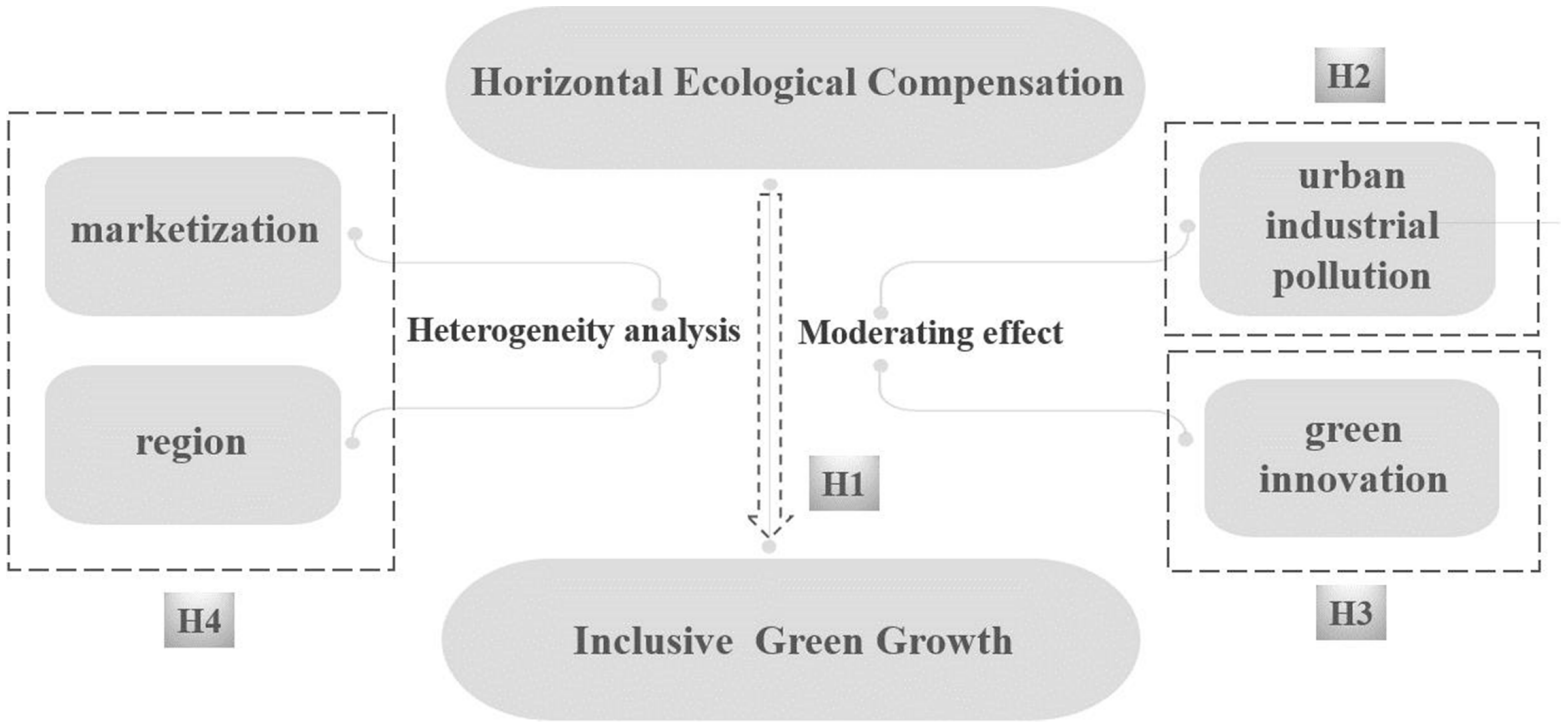
Hypothesis.

The hypothesized mechanism of this article is shown in Figure 2.

## 3 Methodology

### 3.1 Multi-temporal DID baseline regression model

To ensure scientific rigor in evaluating the impact of HEC on IGG, we constructed a regression equation. This method provides a thorough analysis of the policy’s effectiveness. The equation considers the phased implementation of the HEC policy in the Yangtze River Basin, including its expansion to pilot cities and provinces in stages.


(1) $$\begin{array}{l} IGG_{it}=\alpha +\beta HEC\_Policy_{it}+\gamma Control\_Var_{it}\\ \;\;\;\;\;\;\;\;\;\;\;\;\;\;\;+ProFE+YearFE+\varepsilon_{it} \end{array}$$

Among them, i and *t* represent the region and year, respectively, *IGG* represents the value of the IGG index, HEC_Policy, the effect of the HEC policy, and Control_Var represents the set of control variables. ProFE represents the fixed effect of the city, Year_FErepresents the fixed effect of the year, and ε represents the random error term. The estimation coefficient β estimates the average change of the IGG indicator before and after the pilot of the HEC policy.

### 3.2 Moderating effect model

Based on the above analysis, this paper aims to verify the impact of HEC policies on IGG through the moderating effect test:


(2) $$\begin{array}{l} IGG_{it}=\alpha_{2}+\beta_{2}HEC\_Policy_{it}+\eta_{2}HEC\_Policy_{it}\times IPL_{it}\\ +\theta_{2}IPL_{it}+\gamma_{2}Control\_Var_{it}+CiFE+YearFE+\varepsilon_{it} \end{array}$$

(3) $$\begin{array}{l} IGG_{it}=\alpha_{3}+\beta_{3}HEC\_Policy_{it}++\eta_{3}HEC\_Policy_{it}\\ \times GIL_{it}+\theta_{3}GIL_{it}+\gamma_{3}Control\_Var_{it}+CiFE+YearFE+\varepsilon_{it} \end{array}$$

IPL represents the level of urban industrial pollution, and GIL represents the level of green innovation. If $\eta_{2}$ is significant and both $\beta_{2}$ and $\eta_{2}$ have the same sign, it indicates that the level of urban industrial pollution will strengthen the impact of HEC policies on IGG. If $\eta_{3}$ is significant and both $\beta_{3}$ and $\eta_{3}$ are of the same sign, the level of green innovation will strengthen the impact of HEC policies on IGG.

### 3.3 Entropy-weighted TOPSIS method

The entropy-weighted TOPSIS method offers a robust solution to complex multi-attribute decision-making problems, which combines the objective weighting strengths of the entropy weight method with the comprehensive evaluation capabilities of the TOPSIS method. The entropy weight method, grounded in information entropy theory, determines weights by calculating the variation of each evaluation index. This approach minimizes the impact of subjective factors on weight distribution.

The TOPSIS method effectively highlights the relative advantages and disadvantages of evaluation objects. In this study, we construct the initial decision matrix based on the IGG index system. We evaluate data from 87 cities over 14 years, resulting in 1,218 evaluation objects and 22 evaluation indicators. The next step involves establishing the standardized matrix, as detailed below: (n=1218,m=22).


(4) $$Z=\left(\begin{array}{cccc} z_{11} & z_{12} & \cdots & z_{1m}\\ z_{21} & z_{22} & \cdots & z_{2m}\\ \vdots & \vdots & \ddots & \vdots \\ z_{n1} & z_{n2} & \cdots & z_{nm} \end{array}\right)$$

Define maximum:


(5) $$\begin{array}{l} Z^{+}=\left(Z_{1}^{+},\;Z_{2}^{+},\cdots ,\;Z_{m}^{+}\right)\\ =\left(\max\left\{z_{11},\;z_{21},\cdots ,z_{n1}\right\},\max\left\{z_{12},\;z_{22},\cdots ,\;z_{n2}\right\},\max\left\{z_{1m},\;z_{2m},\cdots ,\;z_{nm}\right\}\right) \end{array}$$

Defined minimum:


(6) $$\begin{array}{l} Z^{-}=\left(Z_{1}^{-},\;Z_{2}^{-},\cdots ,\;Z_{m}^{-}\right)\\ =\left(\min\left\{z_{11},\;z_{21},\cdots ,\;z_{n1}\right\},\min\left\{z_{12},\;z_{22},\cdots ,\;z_{n2}\right\},\min\left\{z_{1m},\;z_{2m},\cdots ,\;z_{nm}\right\}\right) \end{array}$$

Define the distance between the *i*th (i=1,2,⋯,n) rating object and the maximum value:


(7) $${D_{i}}^{+}=\sqrt{\overset{m}{\underset{j=1}{\sum }}\omega_{j}{\left(Z_{j}^{+}-z_{ij}\right)}^{2}}$$

Define the distance between the *i*th (i=1,2,⋯,n) rating object and the minimum value:


(8) $${D_{i}}^{-}=\sqrt{\overset{m}{\underset{j=1}{\sum }}\omega_{j}{\left(Z_{j}^{-}-z_{ij}\right)}^{2}}$$

Then this paper can get the unnormalized score of the *i*th (i=1,2,⋯,n) rating object:


(9) $$S_{i}=\frac{D_{i}^{-}}{D_{i}^{+}+D_{i}^{-}}$$

Normalized the score $\left(\overset{n}{\underset{i=1}{\sum }}\tilde{S_{i}}=1\right)$:


(10) $$\tilde{S_{i}}=\frac{S_{i}}{\overset{n}{\underset{i=1}{\sum }}S_{i}}$$

According to the normalized scores, this paper determines the comprehensive evaluation results of 1,218 evaluation objects. By combining the two methods, the entropy-weighted TOPSIS method not only ensures the objectivity of the weights, but also makes full use of the difference information of the original data, making the evaluation results more accurate and reliable.

### 3.4 Variables

#### 3.4.1 Explained variable: IGG

This paper aims to scientifically evaluate the level of IGG by constructing a new index system. Methods used by Dörffel and Schuhmann (53) and Zhou (68) guided us in the creation of the IGG. The system encompasses four dimensions: economic development, social opportunity equity, green production and consumption, and ecological environmental protection.

Economic development is assessed through economic output and income levels. The influence of economic development and living standards on IGG is measured using indicators such as *per capita* GDP and *per capita* disposable income of rural residents.

Social opportunity equity includes educational, medical, and social security opportunities. The distribution and impact of these opportunities on IGG are evaluated through metrics like education expenditure, the number of health technicians per 1,000 population, the number of beds in medical and healthcare institutions per 1,000 population, and the number of participants in urban basic pension and medical insurance.

Sustainable production and consumption are divided into green production and green consumption. Their effects on resources and the environment, as well as their contribution to IGG, are examined through factors like energy consumption, wastewater discharge, and carbon dioxide emissions per unit of output value, as well as *per capita* measurements of these indicators.

Ecological environmental protection focuses on ecological resource endowment and governance. The quality and quantity of ecological resources and the impact of environmental improvement on IGG are assessed through measures such as water resources *per capita*, greening coverage in built-up areas, parkland area *per capita*, wastewater discharge compliance rate, and solid waste utilization rate.

This structured approach aims to provide a comprehensive evaluation of IGG, integrating key aspects of economic, social, and environmental sustainability.

#### 3.4.2 Explanatory variables: HEC policies

This paper investigates the HEC policy using a quasi-natural experiment approach. Cities that adopted the HEC policy serve as the experimental group (coded as 1), while those that did not adopt the policy form the control group (coded as 0). Time dummy variables are used to indicate the periods before (coded as 0) and after (coded as 1) the policy implementation. Since the HEC policy is introduced in stages across different cities, the time dummy variables vary accordingly.

#### 3.4.3 Control variables

Inclusive green growth is influenced by natural, economic, and policy factors. To reduce errors from missing variables and enhance the reliability of our research, this study follows the methodologies of Ren et al. (26), Feng et al. (69), and Wang et al. (70). We selected nine control variables, including science and technology inputs, which are measured by the ratio of science and technology expenditures to regional GDP. Population density is calculated by dividing the total population by the area of the administrative division. The unemployment level is measured by the number of unemployed people divided by the total population of the region.

Water resources *per capita* are assessed by dividing the total water resources of the region by the total population. Human capital is quantified as the natural logarithm of the number of students enrolled in general tertiary institutions per 10,000 people. Sewage discharge *per capita* is measured by the total amount of sewage discharged divided by the total population of the region. Green coverage of built-up areas is determined by the ratio of green coverage to the area of built-up areas, and green space *per capita* in parks is calculated by dividing the total area of green space in parks by the total population of the region.

Missing data were interpolated, and to eliminate the influence of dimensions, the measurement index data of these control variables were standardized.

#### 3.4.4 Mechanism variable: level of green innovation

This paper aims to examine how urban industrial pollution levels and green innovation influence the effectiveness of HEC policies on IGG. Following the research by Yang (67) and Li (71), we measure green innovation by the number of green invention patents granted in each city. To assess urban industrial pollution, we adopt comprehensive approach of Zhang and Hao (72), which includes industrial wastewater emissions, sulfur dioxide emissions, soot emissions, smoke and dust emissions, and nitrogen oxide emissions (Table 1).

**Table 1:** Inclusive green growth index system.

Level 1	Level 2	Level 3
Economic development	Economic output	*Per capita* gdp
		Secondary sector regional production index (previous year = 100)
		Tertiary industry regional production index (previous year = 100)
	Income levels	*Per capita* net income of rural residents (yuan/person)
		*Per capita* disposable income of urban residents (yuan/person)
Social opportunity equity	Equity in educational opportunities	Education expenditure (million yuan)
	Equity of opportunity in healthcare	Health technicians per 1,000 population (persons)
		Number of beds in medical and healthcare institutions per 1,000 population (sheets)
	Equity of opportunity in social security	Number of urban basic pension insurance participants (ten thousand)
		Number of urban basic medical insurance participants at the end of the year (ten thousand)
Sustainable production and consumption	Sustainable production	Energy consumption per unit of output (tonnes/million yuan) = total energy consumption/gdp
		Wastewater discharge per unit of output value (tonnes/million yuan)
		Carbon dioxide emissions per unit of output
		Sulfur dioxide emissions per unit of output
	Consumption	Energy consumption *per capita* (tonnes per person)
		Wastewater discharge *per capita* (tonnes per person)
		Carbon dioxide emissions *per capita* (tonnes per person)
Ecological environmental protection	Ecological resource endowment	Water resources *per capita* (cubic meter)
		Greening coverage in built-up areas (%)
		Parkland area *per capita* (meter)
	Ecological and environmental governance	Wastewater discharge compliance rate (%)
		Integrated solid waste utilization rate (%)

#### 3.4.5 Data source

In this study, we selected data from 85 prefecture-level cities and two municipalities in the Yangtze River Basin, covering the period from 2007 to 2020. The data were sourced from several publications: “China City Statistical Yearbook,” “China Energy Statistical Yearbook,” “China Environmental Statistical Yearbook,” “China Environmental Status Bulletin,” and “Water Resources Bulletin.” Carbon dioxide data were obtained from the CEADs database. Table 2 lists the specific variables used. To handle missing values, we employed an interpolation method.

**Table 2:** Specific variable names and data source.

Variable	Short name	Data source	Data website
Inclusive and Green Growth	IGG	China city statistical year book and China statistical year book on environment	https://cnki.ctbu.edu.cn/CSYDMirror/area/Yearbook/Single/N2021050059?z=D26 and http://cnki.nbsti.net/CSYDMirror/area/Yearbook/Single/N2021070128?z=D26
Green Innovation Level	GIL	Chinese research data services platform	https://www.las.ac.cn/front/dataBase/detail?id=45d5100e2586517c068ee112cdeb7d3a
Urban Industrial Pollution Level	IPL	China industrial enterprises database	https://www.ceicdata.com.cn/en/china/industrial-enterprise
Science and Technology Input	STI	China city statistical yearbook	https://cnki.ctbu.edu.cn/CSYDMirror/area/Yearbook/Single/N2021050059?z=D26
Fixed-Asset Investment	FAI	China city statistical yearbook	
Population Density	PD	China city statistical yearbook	
Unemployment Level	UNE	China city statistical yearbook	
*Per Capita* Water Resources	WR	Water resources bulletin	e.g., https://swj.sh.gov.cn/szy/
Human Capital	HC	China city statistical yearbook	https://cnki.ctbu.edu.cn/CSYDMirror/area/Yearbook/Single/N2021050059?z=D26
*Per Capita* Wastewater Discharge	HWD	China statistical yearbook on environment	http://cnki.nbsti.net/CSYDMirror/area/Yearbook/Single/N2021070128?z=D26
Green Coverage Rate of Built-up Area	GC	China city statistical yearbook	https://cnki.ctbu.edu.cn/CSYDMirror/area/Yearbook/Single/N2021050059?z=D26
*Per Capita* Green Park Area	AGS	China city statistical yearbook	

### 3.5 Study area

The ecological compensation policy in the Yangtze River Basin plays a crucial role in balancing green economic development with ecological construction in the Yangtze River Economic Zone (73). This study investigates the effects of HEC on IGG in the Yangtze River Basin, a pilot region for this policy and one of China’s top practices in recent years. The Yangtze River Economic Belt, a significant economic hub in China, is home to numerous high-quality talent and training bases and boasts one of the highest levels of technological innovation in the country. This study explores the intrinsic mechanism through which the HEC policy on IGG. Our study encompasses 87 cities in the Yangtze River Economic Belt, covering the upper, middle, and lower reaches of the Yangtze River Basin. The upper reaches include Qinghai, Sichuan, the Tibet Autonomous Region, Yunnan, Guizhou, and Chongqing. The middle reaches consist of Hubei, Hunan, and Jiangxi. The lower reaches comprise Anhui, Jiangsu, Zhejiang, and Shanghai.

## 4 Empirical results

### 4.1 Measurement and analysis of IGG

Referring to Zhou’s (68) approach to building an IGG system, this study measured the IGG values of 87 cities in the Yangtze River Basin from 2007 to 2020. The measurement employed the entropy-weighted TOPSIS method, assigning equal importance to each aspect of IGG, with each indicator accounting for 25% of the total weight. Based on these weights, adjustments were made, and the resulting proportions are presented in Table 3.

**Table 3:** Inclusive green growth weight proportions.

Level 1	Level 2	Level 3	Weight
Economic development	Economic output	*Per capita* GDP	9.208
		Secondary sector regional production index	0.047
		Tertiary industry regional production index	0.707
	Income levels	*Per capita* net income of rural residents	7.697
		*Per capita* disposable income of urban residents	7.341
Social opportunity equity	Equity in educational opportunities	Education expenditure	11.892
	Equity of opportunity in healthcare	Health technicians per 1,000 population	6.642
		Number of beds in medical and healthcare institutions per 1,000 population	0.386
	Equity of opportunity in social security	Number of urban basic pension insurance participants	2.937
		Number of urban basic medical insurance participants at the end of the year	3.323
Sustainable production and consumption	Sustainable production	Energy consumption per unit of output	1.748
		Wastewater discharge per unit of output value	4.619
		Carbon dioxide emissions per unit of output	4.12
		Sulfur dioxide emissions per unit of output	1.342
	Consumption	Energy consumption *per capita*	2.216
		Wastewater discharge *per capita*	6.367
		Carbon dioxide emissions *per capita*	4.588
Ecological environmental protection	Ecological resource endowment	Water resources *per capita*	13.845
		Greening coverage in built-up areas	0.421
		Parkland area *per capita*	7.721
	Ecological and environmental governance	Wastewater discharge compliance rate	2.205
		Integrated solid waste utilization rate	0.808

**Figure 3:**
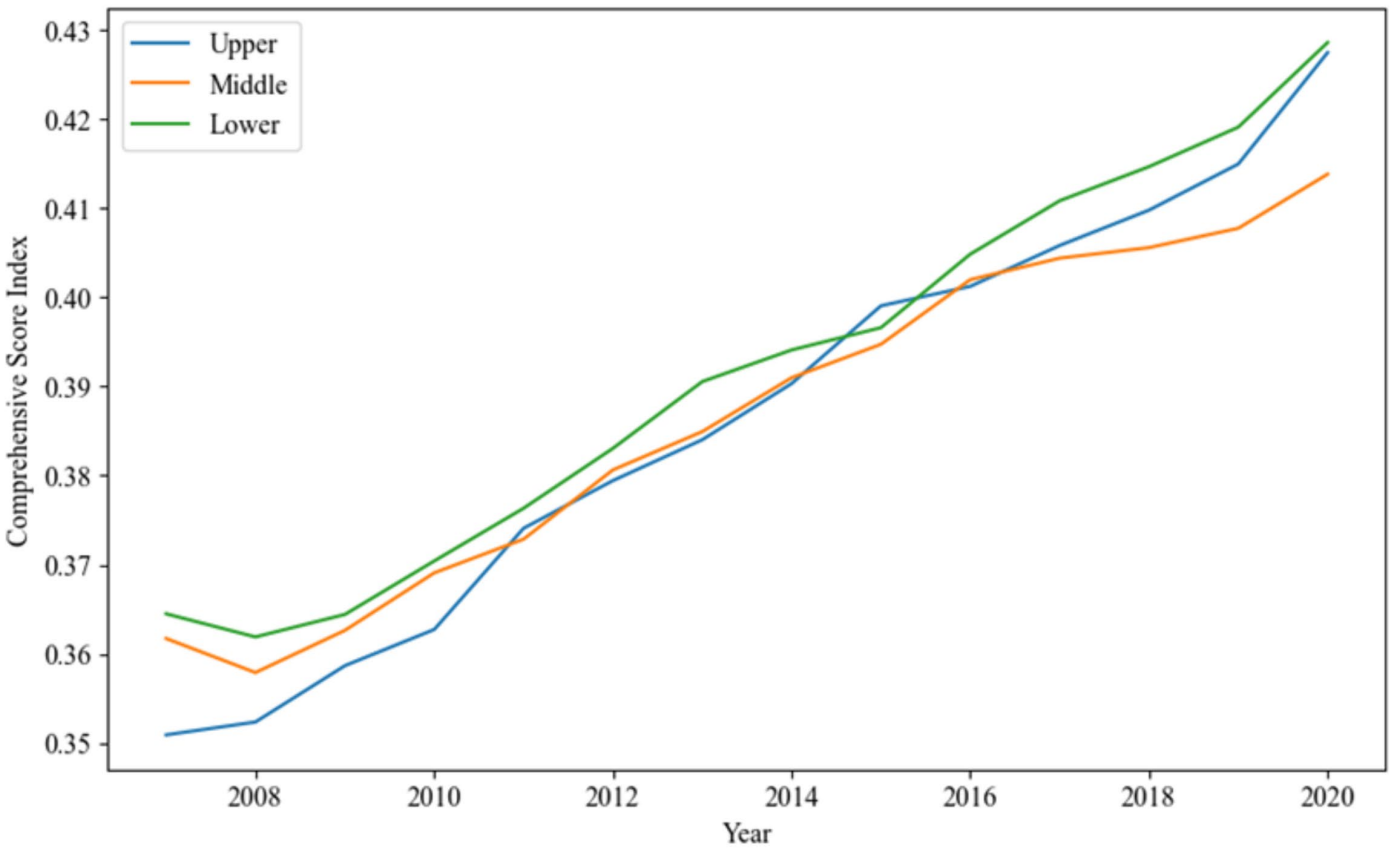
Trend of average comprehensive evaluation score of Yangtze River over Time.

The analysis reveals a mean value of 0.3894, indicating a relatively low level of IGG during the study period. Figure 3 shows that the development trends in the upper, middle, and lower reaches of the Yangtze River generally align with the overall basin trend.

Ecologically, the lower reaches of the Yangtze River exhibit a superior environment compared to the upper and middle reaches. This advantage is attributed to the region’s focus on ecological civilization, green transformation, and development. National initiatives such as the “Ten Principles of Atmosphere,” “Ten Principles of Water,” and “Ten Principles of Soil” have reinforced pollutant discharge standards, improved wastewater discharge compliance, and increased solid waste utilization. Additionally, the “Beautiful China” initiative has enhanced spatial control of ecological protection red lines, permanent basic farmland, urban development boundaries, and other areas, optimizing spatial structure and increasing green spaces. As a result, the lower reaches of the Yangtze River have achieved a relatively high level of green and inclusive growth.

### 4.2 Regression results analysis

The paper utilizes a multi-stage Difference-in-Differences (DID) model to analyze the impact of HEC policies on the IGG level in the Yangtze River Economic Belt. Table 4 presents the findings.

**Table 4:** Regression results.

Variable	(1)	(2)	(3)	(4)
	IGG	IGG	IGG	IGG
HEC_Policy	0.0394*** (23.6012)	0.0175*** (11.2984)	0.3344*** (5.0968)	0.1663*** (3.4529)
STI		−0.0019** (−2.1069)		−0.0719*** (−2.9230)
FAI		0.0029*** (3.1699)		0.0075 (0.1693)
PD		−0.0113* (−1.9154)		−0.4670*** (−3.2204)
UNE		−0.0094*** (−11.9877)		−0.0462** (−2.1414)
WR		0.0152*** (18.5460)		0.4875*** (24.0938)
HC		0.0132*** (4.9545)		−0.2146*** (−3.0669)
HWD		−0.0181*** (−22.0461)		−0.4241*** (−20.2072)
GC		0.0012* (1.8233)		−0.0376** (−2.3486)
AGS		0.0078*** (10.2823)		0.2258*** (12.1077)
Constant	0.4452*** (73.1476)	0.4696*** (14.4821)	0.9592*** (6.0442)	2.1582*** (2.7165)
City	Yes	Yes	Yes	Yes
Year	No	No	Yes	Yes
*N*	1,218	1,218	1,218	1,218
*R* ^2^	0.5374	0.7645	0.7210	0.8547

***p < 0.01, **p < 0.05, *p < 0.1.

First, the estimated coefficient of the policy in column (1) is 0.0394, statistically significant at the 1% level. However, when time fixed effects are considered in column (3), the estimated coefficient of the policy becomes 0. Despite this, the policy effectively promotes IGG, with a statistically significant increase of 0.3344 (*p* < 0.01) after controlling for the time dummy variable. Columns (2) and (4) for provide additional comparison with other control variables, showing policy coefficients of 0.0175 and 0.1663, respectively, both significant at the 1% level.

These results indicate that the HEC policy has a positive impact on IGG in the Yangtze River Economic Belt. The consistent positive regression coefficients across all scenarios suggests that the national HEC policy benefits the IGG of prefecture-level cities in the Yangtze River Basin. Furthermore, the HEC policy shows a significant positive correlation with provincial IGG, even when controlling for city and year effects, thus supporting the H1 hypothesis.

Furthermore, while investment in fixed assets does not significantly promote IGG, investment in science and technology, population density, unemployment level, and *per capita* ecological resource possession are significantly beneficial. Notably, when year effects are not controlled, all control variables significantly increase IGG.

Overall, the analysis demonstrates that the DID coefficients remain significant even after controlling for province and year variables. Both adjusted and unadjusted coefficients are significantly higher, indicating a strong model fit.

### 4.3 Mechanism analysis

Horizontal ecological compensation policies can significantly promote regional IGG (39, 69, 74). These policies influence the emissions of polluting industries, innovation in ecological functional areas, and technological encouragement, which in turn moderate the overall effectiveness of green growth initiatives. Theoretical analyses have demonstrated the efficacy of these policies. To further explore how HEC policies impact IGG, this study substitutes the development variable with the mechanism variable (67, 75) and employs a moderating effect model for testing.

This paper employs the following method to construct the moderating variables: heavily polluting enterprises are significantly impacted by environmental policies, making them a critical focus of these policies. Heavy industry enterprises are major sources of urban pollutants. To avoid environmental penalties, these enterprises respond more proactively to relevant policies, either by reducing pollution emissions or relocating their business. Consequently, the implementation of environmental policies has a more pronounced effect, which moderates the relationship between HEC and IGG (7, 58). Using data from the China Environmental Statistical Yearbook and the China Industrial Enterprises Database, this paper calculates industrial pollutant emissions and assesses industrial pollution in 87 cities in the Yangtze River Economic Belt.

To achieve IGG in cities, technological innovation is essential. This article uses the number of granted urban green invention patents as a measure of green technology innovation. Granted patents, as opposed to applications, more accurately reflect a city’s actual level of technological research and development and offer greater innovation value than utility patents (66, 67). Therefore, this indicator is a rational and feasible measure of green technology innovation.

To investigate the moderating effect of green innovation and industrial pollution level, we conducted a detailed analysis. The results, presented in Table 5, illustrate these relationships.

**Table 5:** Moderating effect test results.

	(1)	(2)
	IGG	IGG
HEC_Policy	0.00594^***^ (0.002)	0.00402^**^ (0.002)

IPL	−0.00235^**^ (0.001)	
	
HEC_Policy× IPL	0.00108^**^ (0.000)	
	
GIL		−0.00172^**^ (0.001)
	
HEC_Policy× GIL		0.00118^**^ (0.001)
	
STI	−0.00019^***^ (0.000)	−0.00018^***^ (0.000)

FAI	0.00000 (0.000)	0.00000 (0.000)

PD	−0.00005^***^ (0.000)	−0.00006^***^ (0.000)

UNE	−0.00194^**^ (0.001)	−0.00196^**^ (0.001)

WR	0.00041^***^ (0.000)	0.00041^***^ (0.000)

HC	−0.00345^***^ (0.001)	−0.00350^***^ (0.001)

HWD	−0.00078^***^ (0.000)	−0.00078^***^ (0.000)

GC	−0.00021^**^ (0.000)	−0.00020^**^ (0.000)

AGS	0.00021^***^ (0.000)	0.00021^***^ (0.000)

cons	0.45230^***^ (0.016)	0.43269^***^ (0.009)

*R* ^2^	0.49614	0.49540
*N*	1218.00000	1218.00000

***p < 0.01, **p < 0.05, *p < 0.1.

Table 5 specifically examines (1) the moderating effect of urban industrial pollution levels. By introducing interaction terms between urban industrial pollution levels and HEC policies into our baseline regression model, we found a positive correlation with IGG at a 5% significance level. This indicates that higher levels of urban industrial pollution amplify the positive impact of HEC policies on IGG. Therefore, hypothesis 2 is confirmed.

To further analyze the moderating effect of green innovation level, we examined (2). By introducing both the green innovation level and its interaction term with the HEC policy into the benchmark regression model, we found that this interaction term is positively correlated with IGG at 5% significance level. This result indicates that green innovation enhances the positive impact of HEC policy on IGG, thereby confirming hypothesis 3.

### 4.4 Parallel trend test and analysis of the dynamic effects

In this study, we apply a multi-period DID model. This approach assumes that both the experimental and control groups follow a consistent trend before the policy implementation, thus meeting the parallel trend hypothesis. Due to the varied timing of policy impacts across cities, using a single year as a reference point for time dummy variables is impractical. Instead, we generate dummy variables based on relative time values specific to each pilot city to effectively implement the HEC policy.


(11) $$\begin{array}{l} IGG_{it}=\beta_{1}Before2_{it}+\beta_{2}Before1_{it}+\beta_{3}Current_{it}\\ \;\;\;\;\;\;\;\;\;\;\;\;\;+\beta_{4}After1_{it}+\beta_{5}After2_{it}+\beta_{6}After3_{it}\\ \;\;\;\;\;\;\;\;\;\;\;\;\;+\beta_{7}After4_{it}+\beta_{8}After5_{it}+\beta_{9}After6_{it}\\ \;\;\;\;\;\;\;\;\;\;\;\;\;+\gamma Control\_Var_{it}+ProFE+YearFE+\varepsilon_{it} \end{array}$$

**Figure 4:**
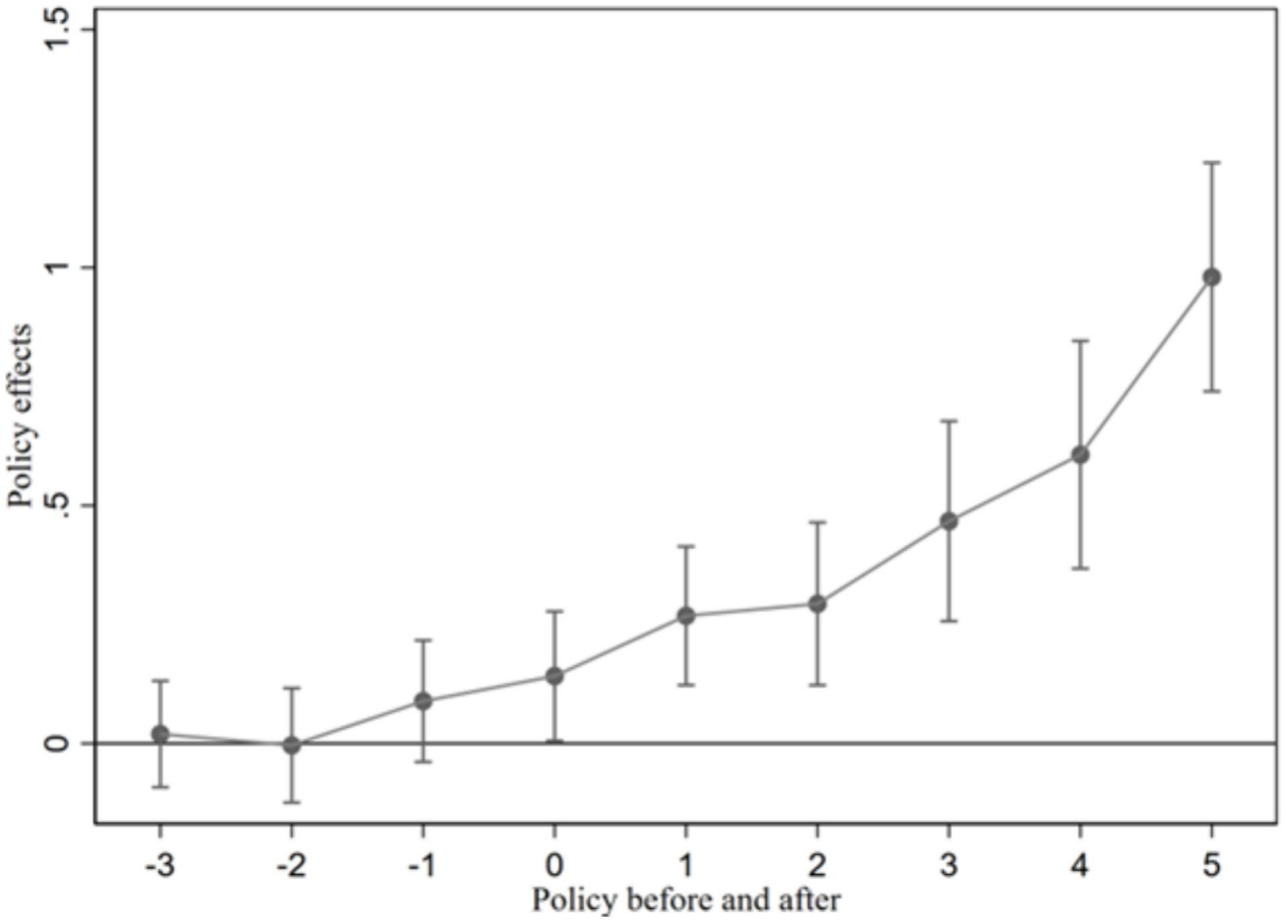
Parallel trend test results.

The time dummy variable captures the observed values of prefecture-level cities before, during, and after the implementation of the HEC policy over several years. For cities that are not pilot cities, these dummy variables are set to 0. Figure 4 illustrates the results. Before the policy, the coefficients of the relative time dummy variables are not statistically significant and have small values. This result indicates no significant difference in IGG between the experimental and control group before the policy, which also supports the parallel trend hypothesis.

The policy’s dynamic impact is evident within 2 years of implementation, showing a gradual influence on IGG. After 2 years, the impact coefficient of the HEC policy demonstrates a significant and consistently increasing positive trend. This suggests that the pilot policy effectively promotes IGG in prefecture-level cities along the Yangtze River, albeit with some delay.

### 4.5 Robustness test: placebo test

This article has controlled for numerous urban characteristic variables in a quasi-natural experiment. However, some unobserved factors might still affect the assessment results of the HEC policy. In the multi-period DID analysis, the timing of policy implementation varies among pilot cities. Therefore, it is essential to conduct a placebo test by randomly generating pseudo-treatment group variables and pseudo-policy shock variables. This involves selecting random sample periods for each sample object as its policy time.

**Figure 5:**
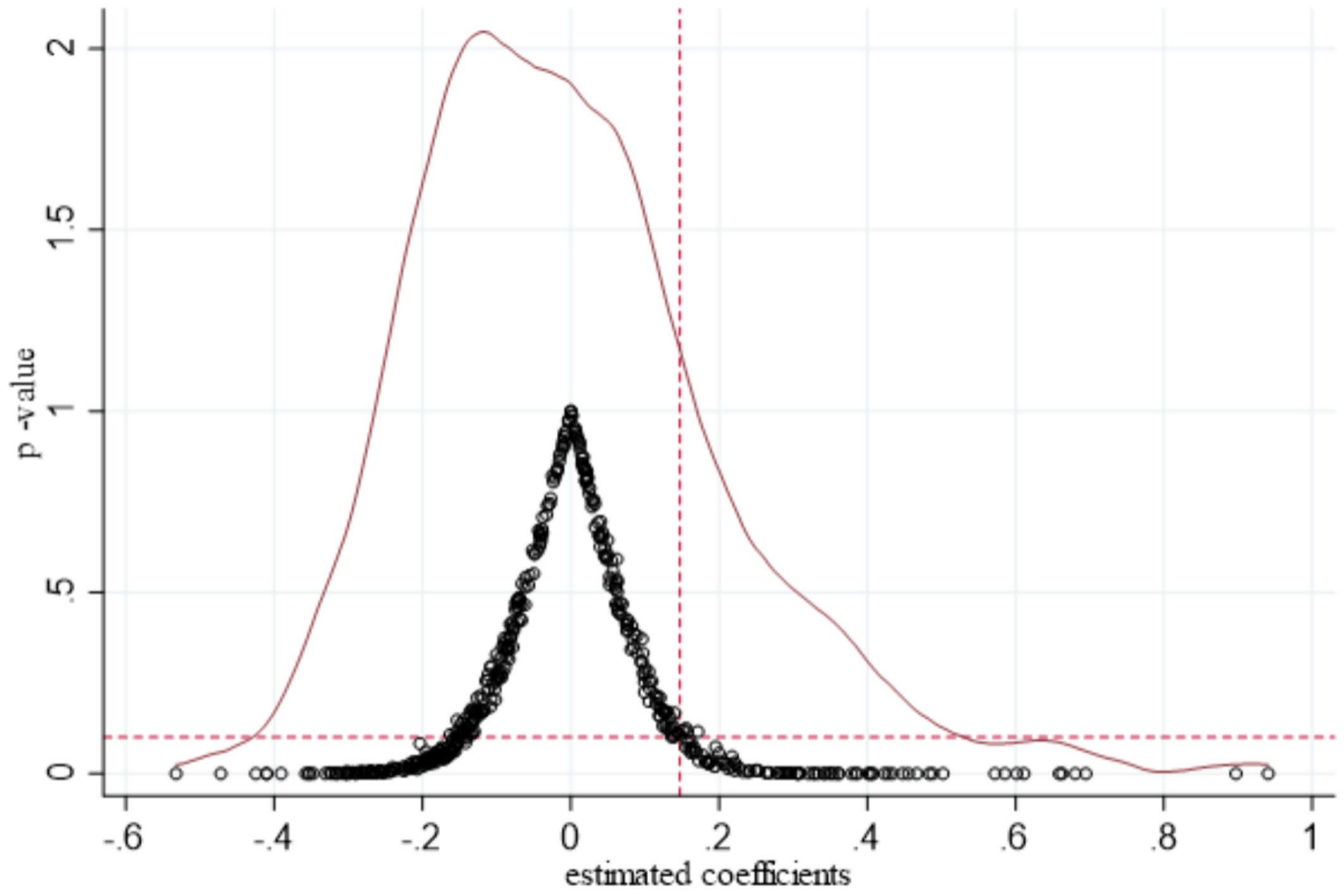
Placebo test.

To achieve this, we randomly sampled 87 prefecture-level cities and municipalities directly under the central government, ensuring no duplication of experimental provinces and policy time points. During each sampling, 70 cities were selected as virtual pilot areas, while the remaining 17 cities were designated as virtual non-pilot areas. This random sampling process was repeated 500 times, followed by regression analysis. We obtained 500 sets of simulated variables and illustrate the distribution of the 500 kernel densities and their values in Figure 5.

The vertical dotted line marks a coefficient of 0.1663, which represents the baseline regression in this study. Our analysis found that the actual regression coefficient is at the lower end of the permutation test distribution and is below 0.1, which indicates a significant deviation from the placebo test results. Most estimated coefficient values, however, are above 0.1, and the mean regression coefficient from random sampling is near zero. These findings suggest that our baseline regression result is robust and passes the placebo test, supporting the reliability of our study’s evaluation.

### 4.6 Heterogeneity analysis

Given the varying marketization processes across cities, it is essential to integrate both government and market mechanisms in HEC policies. Based on the marketization index, the 87 cities were divided into three groups: low (0–5), medium (5–7), and high (above 7) marketization levels. This classification, derived from the average total marketization score from 2007 to 2020, was used to assess how pilot policies for HEC impact IGG in cities with different marketization levels.

Table 6 presents the estimated results for the impact of heterogeneous marketization on IGG. The findings indicate that HEC policies have a minimal effect in cities with low marketization, a slight positive impact in cities with medium marketization, and a significant positive impact in cities with high marketization. As urban marketization levels increase, information asymmetry diminishes, allowing market supply and demand to be more accurately reflected. This enables the government to develop and implement more effective and targeted policies, supporting the confirmation of Hypothesis 4.

**Table 6:** Heterogeneous result of marketisation.

	(1)	(2)	(3)
	Low	Medium	High
*HEC_Policy*	0.000 (.)	0.080 (1.29)	0.122* (1.90)
*N*	70	745	354
*R* ^2^	0.960	0.898	0.952
Adj. *R*^2^	0.90	0.88	0.93

This article aims to explore whether different regions along the Yangtze River will produce varying effects on IGG. The cities are categorized into urban clusters in the upper, middle, and lower reaches of the Yangtze River, based on their respective provinces. The objective is to investigate whether the initial implementation of HEC policies have different effects in various regions. The estimated results are presented in Table 7.

**Table 7:** Heterogeneous result of regions along the Yangtze River.

	(1)	(2)	(3)
	Upper	Middle	Lower
*HEC_Policy*	0.130 (1.34)	−0.065 (−1.14)	0.179** (2.00)
*N*	476	266	476
*R* ^2^	0.787	0.944	0.864
Adj. *R*^2^	0.76	0.93	0.85

The paper also examines whether the effects of HEC policies vary across different regions of the Yangtze River. Cities are categorized into urban clusters in the upper, middle, and lower reaches of the river, according to their respective provinces. Table 7 shows the estimated results for this regional analysis.

The findings reveal that the HEC policy does not positively influence IGG in the middle reaches of the Yangtze River. In contrast, HEC has significant positive effects in urban areas of both the upper and lower reaches. This regional variation may be due to differences in the timing and intensity of policy implementation across provinces. The upper and lower reaches of the Yangtze River, being crucial areas and initial pilot areas for these policies, benefit from substantial government investment and social attention, which enhances the positive impact on IGG.

## 5 Conclusion

This article uses the IGG Index to measure and analyze the level of green development and regional differences in the Yangtze River Basin from 2007 to 2020. The findings indicate that the mean value of IGG in this period is 0.3894, suggesting a low overall level and highlighting the need for further improvement.

The empirical analysis demonstrates that the HEC policy has significantly enhanced the overall level of IGG in cities within the Yangtze River Basin. This policy also shows lag and dynamic effects, and its reliability is confirmed through a placebo test.

Additionally, the moderating effect test reveals that urban industrial pollution and green technology innovation significantly influence the impact of the HEC policy on IGG. These factors are crucial in how the policy affects green growth levels.

Heterogeneity analysis shows that the policy’s impact varies based on the degree of marketization in different cities. As urban marketization increases, the positive impact of the policy becomes more significant. Furthermore, the influence of HEC on IGG differs across cities in the upper, middle, and lower reaches of the Yangtze River. The positive effects are more pronounced in cities in the upper and lower reaches.

Overall, this analysis indicates that national strategic planning and policy measures have a substantial impact on green development in the Yangtze River Basin.

Our research fundamentally contributes to understanding the complex interplay between inclusive urban expansion and ecological conservation. By examining the nexus between ecological compensation policies and IGG, we enrich the scholarly discourse on green urban development. We contend that our findings provide valuable insights for addressing regional disparities and advancing inclusive urban development.

Firstly, due to the lagging and dynamic effects of HEC policies, we recommend increasing both the investment and duration of these policies to continuously enhance the level of IGG in cities along the Yangtze River Basin. It is also essential to devise precise compensation policies tailored to each context. The effectiveness of marketization varies across cities; highly marketized areas may favor market-based approaches, while less marketized regions might benefit more from government-led or socially participatory mechanisms.

Moreover, building model cities for HEC in the lower Yangtze River and strengthening policies for the upper and middle reaches is suggested. Priority should be given to implementing these policies in cities polluted by heavy industries, with strengthened supervision of heavy polluters to accelerate policy implementation and gradually expand the scope of the pilot programs.

Supporting green technology innovation requires fortifying intellectual property rights mechanisms and cracking down on infringement. Additionally, establishing collaborative platforms and enhancing market demand are crucial for advancing environmentally sustainable technological solutions.

Despite these contributions, our paper has limitations that future research can address. Firstly, while the Yangtze River Basin is a significant pilot area for HEC, other basins, such as the Yellow River Basin, also warrant attention. Future studies could expand the scope to include these areas, enhancing the universality of data and generalization of conclusions. Secondly, regarding the influence mechanisms, future research should consider improving variable selection to study the impact of HEC on IGG from a more comprehensive perspective.

## Data Availability

Publicly available datasets were analyzed in this study. This data can be found here: https://www.stats.gov.cn.
